# Characterisation of the *in vitro* activity of a Nitazoxanide-*N*-methyl-1*H*-benzimidazole hybrid molecule against albendazole and nitazoxanide susceptible and resistant strains of *Giardia intestinalis* and its *in vivo* giardicidal activity

**DOI:** 10.1590/0074-02760190348

**Published:** 2020-02-07

**Authors:** Félix Matadamas-Martínez, Benjamín Nogueda-Torres, Rafael Castillo, Alicia Hernández-Campos, María de la Luz Barrera-Valdes, Gloria León-Ávila, José Manuel Hernández, Lilián Yépez-Mulia

**Affiliations:** 1 Universidad Nacional Autónoma de México, Facultad de Química, Departamento de Farmacia, Mexico City, Mexico; 2 Escuela Nacional de Ciencias Biológicas-Instituto Politécnico Nacional, Departamento de Parasitología, Mexico City, Mexico; 3 Escuela Nacional de Ciencias Biológicas-Instituto Politécnico Nacional, Departamento de Zoología, Laboratorio de Genética, Mexico City, Mexico; 4 >Centro de Investigación y de Estudios Avanzados del Instituto Politécnico Nacional, Departamento de Biología Celular, Mexico City, Mexico; 5 Instituto Mexicano del Seguro Social, Centro Médico Siglo XXI, Unidad de Investigación Médica en Enfermedades Infecciosas y Parasitarias, Hospital de Pediatría, Mexico City, Mexico

**Keywords:** giardicidal activity, hybrid molecule, resistant strains, cytoskeletal proteins, *in vivo* activity

## Abstract

**BACKGROUND:**

It was previously demonstrated that **CMC-20,** a nitazoxanide and *N*-methyl-1*H*-benzimidazole hybrid molecule, had higher *in vitro* activity against *Giardia intestinalis* WB strain than metronidazole and albendazole and similar to nitazoxanide.

**OBJETIVES:**

To evaluate the *in vitro* activity of **CMC-20** against *G. intestinalis* strains with different susceptibility/resistance to albendazole and nitazoxanide and evaluate its effect on the distribution of parasite cytoskeletal proteins and its *in vivo* giardicidal activity.

**METHODS:**

**CMC-20** activity was tested against two isolates from patients with chronic and acute giardiasis, an experimentally induced albendazole resistant strain and a nitazoxanide resistant clinical isolate. **CMC-20** effect on the distribution of parasite cytoskeletal proteins was analysed by indirect immunofluorescence and its activity was evaluated in a murine model of giardiasis.

**FINDINGS CMC-20:**

showed broad activity against susceptible and resistant strains to albendazole and nitaxozanide. It affected the parasite microtubule reservoir and triggered the parasite encystation. In this process, alpha-7.2 giardin co-localised with CWP-1 protein. **CMC-20** reduced the infection time and cyst load in feces of *G. muris* infected mice similar to albendazole.

**MAIN CONCLUSIONS:**

The *in vitro* and *in vivo* giardicidal activity of **CMC-20** suggests its potential use in the treatment of giardiasis.

*Giardia intestinalis* is a flagellate protozoan that causes giardiasis, a diarrheal disease that affects human worldwide. *G. intestinalis* comprises eight different assemblages (A-H); from them, assemblages A and B are considered zoonotic since they infect both humans and domestic animals. Assemblage B is more commonly associated with human infections than assemblage A (58% and 37%, respectively).^1^ *Giardia* infection is an important cause of morbidity and due to its socioeconomic significance was included in the World Health Organization (WHO) Neglected Disease Initiative.^2^ It has been associated with intestinal dysbiosis and the subsequent development of chronic post-infection inflammatory bowel syndrome.^2^ In 2010, the World Health Organization (WHO) reported that *Giardia* caused 28.2 million cases of foodborne disease and 26,270 disability-adjusted life years.^3^ In the United States, approximately 20,000 new cases of giardiasis are reported annually; however, from 2009 to 2010, the number of reported cases of giardiasis increased 1.9%.^4^ Importantly, migration from endemic places and an increase international travelling are changing the epidemiology of giardiasis in developed countries. In Sonora State (Norwest of Mexico) giardiasis prevalence between 20 to 30% was determined in the childhood population.^5^ The ingestion of contaminated food and water with parasite cysts represents the main transmission route of giardiasis. Most infections in humans are asymptomatic, but in some cases acute or chronic disease are associated with severe diarrhea, malabsorption and weight loss. The trophozoite stage is the dividing and metabolically active form that is the main cause of the pathogenesis observed during giardiasis. The trophozoite adhesion to epithelial cells is an important step in the pathogenesis process and it is mainly mediated by the ventral disc. The ventral disc, median body, funis, eight motile flagella and axonemes, are constituted by the alpha and beta tubulin heterodimer, the structural unit of microtubules that together with giardins and actin microfilaments form the parasite cytoskeleton. The complex parasite cytoskeleton is essential for cell division, motility, attachment, intracellular transport and encystation/excystation process.^6^

Metronidazole (MTZ) is the drug of choice for the treatment of giardiasis, however, it causes severe secondary effects and treatment failure as well as parasite resistance have been reported.^7^ Albendazole (ABZ), a benzimidazole derivative, is alternatively used in the treatment of giardiasis. It disrupts *Giardia* ventral disc organization.^8^ A meta-analysis of the effectiveness of ABZ compared with MTZ as treatments for *G. intestinalis* infections showed that ABZ (400 mg/day for five days) was as effective as MTZ. Importantly, patients treated with ABZ had fewer side effects in comparison to those treated with MTZ.^9^ Alternatively, nitazoxanide (NTZ), a 5-nitrothiazolyl derivative, is also used to treat giardiasis. In clinical trials, NTZ´s efficacy varies between 71 to 100% and it is less toxic than MTZ.^10^ However, parasite resistance to NTZ and ABZ has been experimentally induced^11^ ^,^ ^12^ and it has been reported in clinical isolates.^13^ ^,^ ^14^ Therefore, efforts have been focused on the development of new giardicidal molecules. In this regard, Navarrete-Vazquez et al.^15^ synthesised a benzologue of NTZ against *G. intestinalis* (IC_50_ = 0.297 µM). It was four more active than NTZ (IC_50_ = 1.214 µM) and 18 times more active than MTZ (IC_50_ = 5.35 µM), respectively. Recently, we reported the synthesis and the *in vitro* giardicidal activity of a novel nitazoxanide and *N*-methyl-1*H*-benzimidazole hybrid molecule, named **CMC-20**. It showed against *G. intestinalis* WB strain an IC_50_ value of 0.010 μM, lower than the IC_50_ values of ABZ and MTZ and similar to NTZ. The proteomic analysis of **CMC-20** treated trophozoites revealed significant changes in the expression level of alpha and beta tubulin, as well as some proteins that participate in the encystment process.^16^

Since **CMC-20** showed potent activity against WB strain, a reference strain susceptible to ABZ and NTZ, our interest was to evaluate its giardicidal activity against parasite strains susceptible or resistant to these reference drugs. These strains included an isolate from a patient with chronic giardiasis and one from a patient with acute giardiasis; an experimentally induced ABZ resistant strain and a NTZ resistant clinical isolate. Besides, it was analysed by indirect immunofluorescence (IFI) the effect of **CMC-20** on the distribution of cytoskeletal proteins such as beta tubulin and alpha tubulin, as well as acetylated, tyrosinated and detyrosinated-alpha tubulin and other cytoskeletal proteins such as alpha-7.2 giardin and actin. Finally, its giardicidal activity was determined in a murine model of giardiasis in comparison to ABZ.

## MATERIALS AND METHODS

*Chemistry* - The benzimidazole derivative **CMC-20** was prepared as previously described by our group.^16^ The chemical formula of **CMC-20** is presented as Supplementary data.

*Parasites* - For susceptibility assays the following *G. intestinalis* strains were used: WB strain (ATCC # 30957), a reference strain susceptible to ABZ and NTZ; isolates from patients with chronic (IMSS-1090-1) or acute (UNAM-0688-2) giardiasis,^17^ an experimentally induced ABZ resistant strain (BRIS/91/HEPU/1411)^18^ and a NTZ resistant clinical isolate (N1-INP).^13^ All the parasite strains were genotyped as assemblage AI.

*Susceptibility assays* - **CMC-20**, ABZ and NTZ at 26.1, 37.6 and 32.5 mM, respectively, were previously dissolved in dimethyl sulfoxide (DMSO) followed by drug dilution in *Giardia* culture medium, making a final stock solution of 26.1, 37.6 and 32.5 µM, respectively. Further dilutions were made in order to obtain the following working concentrations: 0.013 - 1.30 µM of **CMC-20**; 0.018 - 1.88 μM of ABZ and 0.016 - 1.62 µM of NTZ. The maximum DMSO concentration in cultures was lower than 0.001%, a concentration that does not affect *G. intestinalis* growth *in vitro*.

Parasites were cultured in TYI-S-33 modified medium supplemented with 10% calf serum and bovine bilis. Trophozoites (5 × 10^4^/mL) were incubated with the drugs at the different concentrations for 48 h at 37ºC. Trophozoites with DMSO (at the highest concentration used in working solutions) were included as negative control. At the end of the treatment trophozoites were washed and subcultured for another 48 h in fresh medium free of drugs. Parasites were counted with a hemocytometer and the concentration that inhibits the trophozoite growth by 50% (IC_50_) was calculated by Probit analysis. Three independent assays were performed by triplicate. For each assay, drugs were dissolved in DMSO and working solutions were newly prepared as already mentioned.

*Cytotoxicity assay* - Madin-Darby canine kidney cell line (MDCK) was used to assess the cytotoxic effect of ABZ and NTZ as previously described for **CMC-20**.^16^ The cells were cultured in standard culture medium [MEM with 10% foetal bovine serum (FBS)]. The final concentration of DMSO in the culture medium remained below 0.01%. The cultures were incubated with ABZ and NTZ at different concentrations for 24 h at 37ºC, 5% CO_2_ and 95% relative humidity. Untreated cells were included as controls. Cytotoxicity was determined using the colorimetric MTT assay. Viability percentage was calculated with respect to control cells and 50% cytotoxicity concentration (CC_50_) was estimated using the GraphPad Prism (4.0 version). The assay was done by triplicate.

*Production of antibodies* - Antibodies against recombinant actin and alpha-7.2 giardin proteins were produced in BALB/c mice meanwhile antibodies against CWP-1 were produced in Wistar rats. Animals were injected intraperitoneally with 100 μg recombinant proteins at days 0, 15 and 30. The first boost was done with 10:1 protein/ Complete Freund´s adjuvant mixture (Sigma-Aldrich), and the following boosts were done with 1:1 protein/Incomplete Freund´s adjuvant mixture, (Sigma-Aldrich). Immune sera were obtained four days after the last boost and assayed by enzyme-linked immunosorbent assay (ELISA).

*IFI assays* - For this purpose, trophozoites of *G. intestinalis* WB strain (2 X 10^6^) were incubated for 24 h in culture medium with **CMC-20** at the same concentration (7.8 µM) previously used for proteomic studies.^16^ Trophozoites without treatment were included as negative control. Control and **CMC-20** treated parasites were placed on poly-L-lysine coated glass slides (Sigma-Aldrich) and incubated for 10 min to allow cells to attach to the glass surface. Then, parasites were fixed with 4% paraformaldehyde in phosphate-buffered saline (PBS) pH 7.2 for 15 min and washed twice with PBS. The parasites were permeabilised with 0.5% Triton X-100-SDS for 10 min and washed twice with PBS, and nonspecific binding was blocked with 1% bovine serum albumin (BSA) in PBS for 1 h at room temperature. After this, parasites were incubated at room temperature for 1h with the following monoclonal antibodies: anti-alpha tubulin clone B-512 (Sigma-Aldrich) (1:1000), anti- acetylated tubulin clone 6-11B-1 (Sigma-Aldrich) (1:1000), anti-tyrosinated tubulin clone TUB-1A2 (Sigma-Aldrich) (1:100), anti-detyrosinated tubulin clone YOL1/34 (Millipore)(1:100), anti-beta tubulin clone KMX-1(Millipore) (1:500), and polyclonal antibodies against either alpha-7.2 giardin (1:1000), actin (1:500) or CWP-1 (1:1000). Secondary antibodies anti-mouse-FITC and anti-rat-TRITC (Millipore) (1:500) were incubated for 1h at room temperature. The cover slips were washed three times in PBS and mounted on glass slides with Vectashield (Vector Laboratories). The images were obtained using a Leica confocal microscope and epifluorescence (Leica DMIRE2 and Olympus).

*Evaluation of the in vivo giardicidal activity of CMC-20* - The biological activity of CMC-20 was evaluated against *G. muris* infection in BALB/c mice. ABZ was included as the reference drug. For this, groups of 6 female BALB/c mice aged four-six weeks old were each orally infected with 1 x 10^3^ cysts of *G. muris* suspended in 0.2 mL PBS, pH 7.2*.* CMC-20 and ABZ at 50 mg/kg/day were administered intragastrically at days seven and eight post-infection (pi). Animals infected but not treated were included as negative control. Feces excreted from control group and treated groups were collected for over a 2 h period daily from tree to 35 day after infection. Cysts were purified from feces using a sucrose gradient and the number of shed cysts was counted using a hemocytometer. Animal experiments were performed according to the Norma Oficial Mexicana (NOM-062-Z00-1999) published on August 22, 2009.

## RESULTS

*Activity of CMC-20 against G. intestinalis strains* - The activity of CMC-20 and the reference drugs, ABZ and NTZ, against all the parasite stains tested is presented in Table. ABZ was active against WB strain and isolates from patients with chronic (IMSS-1090-1) or acute (UNAM-0688-2) giardiasis (IC_50_ of = 0.047, 0.040 and 0.034 µM, respectively), however, it was more active against the isolate from a patient with acute giardiasis. NTZ was more active against both isolates (IC_50_ = 0.009 µM) than against WB strain (IC_50_ = 0.018 µM). In relation to CMC-20, it was active against the isolates and WB strain, however, it was more active against WB strain (IC_50_ of 0.015 µM) than against the isolates from patients with chronic and acute giardiasis (IC_50_ = 0.052 and 0.033 µM, respectively). CMC-20 was 1.6 times more active against the acute giardiasis isolate in comparison to the chronic giardiasis isolate. In general, NTZ was 5.8 and 3.7 times more active than CMC-20 and 4.4 and 3.8 times more active than ABZ against the isolates from patients with chronic and acute giardiasis. However, CMC-20 was three times more active than ABZ against WB strain (IC_50_ = 0.015 µM vs 0.047 µM), but similar to NTZ (IC_50_ = 0.015 µM vs 0.018 µM).

In relation to the ABZ resistant strain, **CMC-20** was three times more potent than ABZ (IC_50_ = 0.045 µM vs 0.137 µM); but showed half the activity of NTZ (IC_50_ = 0.045 µM vs 0.021 µM). Regarding the NTZ resistant clinical isolate, **CMC-20** was 28 times more active than NTZ (IC_50_ = 0.071 vs 1.97 µM) and 3.9 times more active than ABZ (IC_50_ = 0.071 vs 0.277 µM).

**TABLE:** Activity (IC_50_ μM) of Albendazole (ABZ), nitazoxanide (NTZ) and **CMC-20** against susceptible and resistant strains of *Giardia intestinalis* and selectivity index* (SI)

Compound	WB strain	IMSS-1090-1 isolate	UNAM-0688-2 isolate	rABZ strain	rNTZ isolate
ABZ SI	0.047 ± 0.001 800	0.040 ± 0.004 940	0.034 ± 0.003 1105	0.137 ± 0.01 274	0.277 ± 0.001 136
NTZ SI	0.018 ± 0.005 1805	0.009 ± 0.005 3611	0.009 ± 0.005 3611	0.021 ± 0.007 1547	1.973 ± 0.2 16
CMC-20 SI	0.015 ± 0.002 1733	0.052 ± 0.003 500	0.033 ± 0.004 787	0.045 ± 0.004 577	0.071 ± 0.004 366

rABZ: an induced albendazole resistant strain; rNTZ: nitazoxanide resistant clinical isolate. *The selectivity index (SI) of **CMC-20**, ABZ and NTZ was calculated as the ratio of cytotoxicity to biological activity (SI = CC_50_ MDCK cells/IC_50_ strains).

*Cytotoxicity assays* - The cytotoxicity of **CMC-20**, ABZ and NTZ was tested on Madin-Darby canine kidney cell line (MDCK) and their CC_50_ values were > 26 µM,^16^ > 37.6 µM and > 32.5 µM, respectively. To calculate their selectivity index (SI) against the parasite strains, CC_50_ values of 26 µM, 37.6 µM and 32.5 µM of **CMC-20**, ABZ and NTZ, respectively, were used (Table). NTZ had the highest SI against the clinical isolates and the ABZ resistant strain. However, the SI of **CMC-20** was higher than NTZ against the NTZ resistant clinical isolate and its SI against WB strain was close to NTZ. In addition, **CMC-20** showed higher SI than ABZ against WB strain and the ABZ and NTZ resistant isolates.

**Fig. 1:**
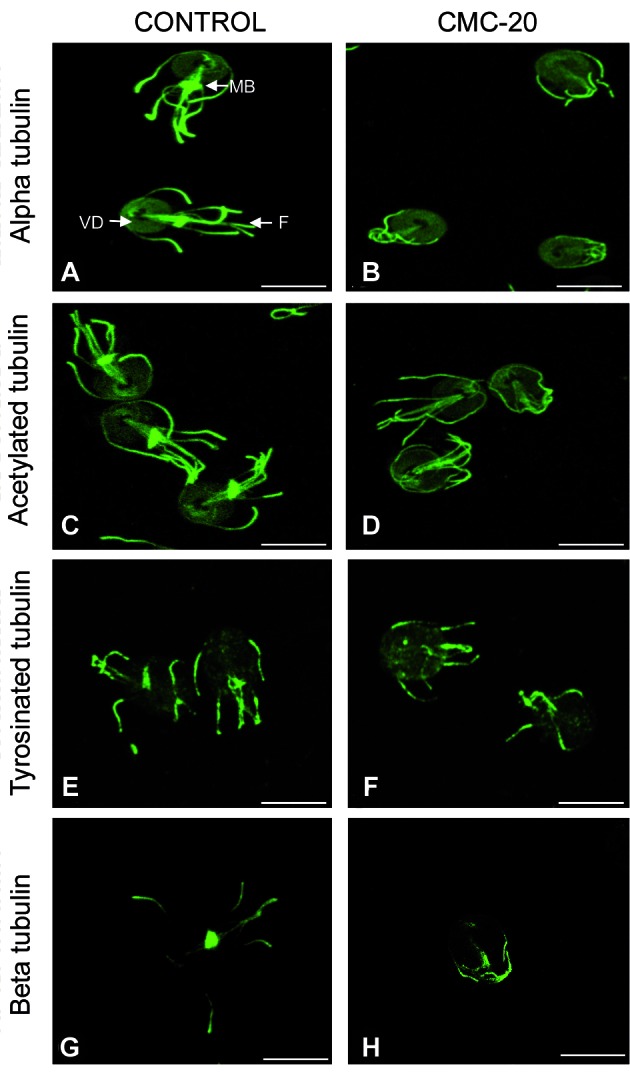
distribution of parasite cytoskeletal proteins in control (A,C,E,G) and **CMC-20** treated trophozoites (B,D,F,H) analysed by indirect immunofluorescence (IFI) using a set of specific antibodies against cytoskeletal proteins. In control parasites the presence of alpha tubulin (A) and acetylated-alpha tubulin (C) was observed mainly in the ventral disc, flagella and median body. Tyrosinated-alpha tubulin (E) was localised in the cytoplasm, flagella and in the median body of control parasites. In addition, beta tubulin (G) was observed in the flagella and in the median body of control parasites. In **CMC-20** treated parasites, cytoskeletal proteins were localised in the same structures as in control parasites, with the exception of median body. Trophozoites treated with **CMC-20** lose their characteristic pear shape and in some cells flagella retraction was observed (VD: ventral disc; MB: median body; F: flagella; Bar: 5 μm).

*Analysis of the effect of CMC-20 on the distribution of cytoskeletal and encystation proteins by IFI* - In this study the effect of CMC-20 on the distribution of different cytoskeletal parasite proteins was analysed by IFI. These assays showed the presence of alpha tubulin and acetylated-alpha tubulin in the ventral disc, flagella, basal bodies and median body of control WB trophozoites (Fig. 1A, C). In CMC-20 treated parasites, alpha tubulin and acetylated-alpha tubulin were recognised in the same structures as in control, with the exception of the median body. In addition, major morphological changes and some rounded cells with retraction of the flagella were observed (Fig. 1B, D). The presence of tyrosinated-alpha tubulin, using the antibody TUB-1A2, was observed in the cytoplasm, flagella and median body of control parasites (Fig. 1E). In CMC-20 treated parasites, tyrosinated-alpha tubulin was recognised in the same structures as in control with the exception of the median body (Fig. 1F). However, no recognition of detyrosinated-alpha tubulin was observed in both control and treated parasites.

**Fig. 2:**
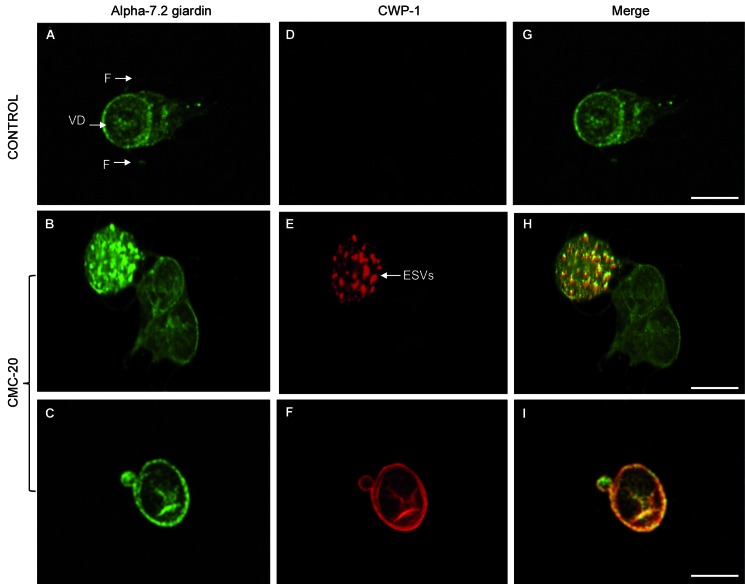
localisation of alpha-7.2 giardin and CWP-1 in control and **CMC-20** treated parasites. Alpha-7.2 giardin was localised in association with the plasma membrane, ventral disc and slightly on flagella of control trophozoites (A, G), however, it was observed in vesicle-like structures (B) and on the surface of **CMC-20** treated parasites (C). Control parasites did not stain for CWP-1 (D). CWP-1 localised in ESVs and on the surface of **CMC-20** treated parasites (E, F). Alpha-7.2 giardin co-localised with CWP-1 both in ESVs and on the cell surface of treated parasites (H and I). VD: ventral disc; F: flagella; ESVs: encystation specific vesicles; Bar: 5 μm.

**Fig. 3:**
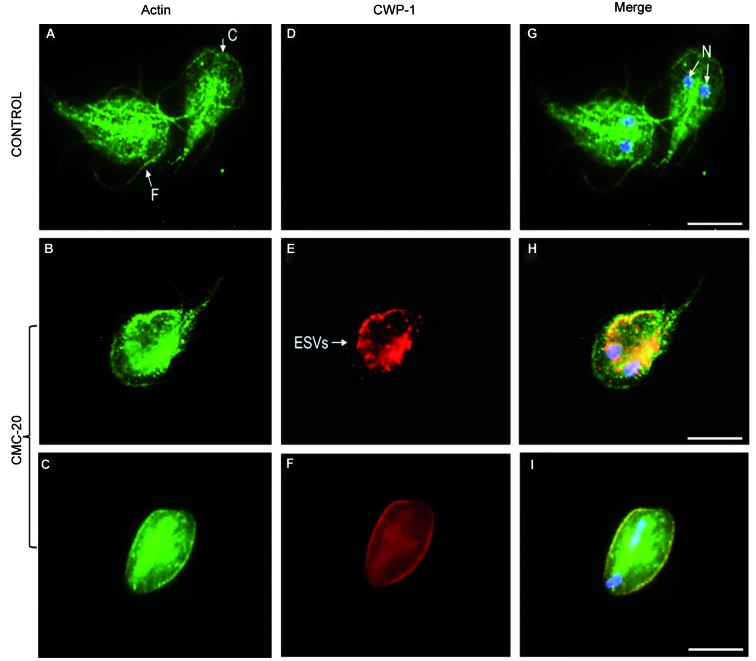
actin localisation in control and **CMC-20** treated parasites. Actin localised to the cortex, flagella and around nuclei of control parasites (A, G). Control trophozoites did not stain for CWP-1 (D). In **CMC-20** treated parasites actin was detected in the caudal flagella, the cortex and as condensed patches in the cytoplasm (B). It was also detected on the surface of cysts (C). CWP-1 was detected in ESVs (E) and on the surface of cysts (F). Co-localisation of CWP-1 with actin was observed (H, I). The nuclei are stained with DAPI (blue). F: flagella; C: cortex; N: nuclei; Bar: 5 μm.

Regarding beta tubulin, the flagella and median body of control parasites were recognised using the antibody KMX-1 (Fig. 1G). However, the median body was not stained for beta tubulin in **CMC-20** treated parasites (Fig. 1H). The no recognition of the median body by antibodies anti-alpha and anti- beta tubulin was observed in 80% of the treated cells. Alpha-7.2 giardin was localised in association with the plasma membrane, ventral disc and slightly on flagella of control parasites (Fig. 2A). Nevertheless, in **CMC-20** treated parasites, alpha-7.2 giardin was evidenced on the surface of cyst-like cells as well as in vesicle-like structures (Fig. 2B, C). The use of an anti-CWP-1 antibody confirmed that the rounded cells were encysted parasites and the vesicle-like structures were encystation specific vesicles (ESVs) (Fig. 2E, F). About 10% of the treated trophozoites were transformed into cysts. Control parasites did not stain for CWP-1 (Fig. 2D, G). Interestingly, it was evident that in **CMC-20** treated parasites, CWP-1 co-localised with alpha-7.2 giardin (Fig. 2H, I). Actin was detected in the cortex, flagella and around nuclei of control trophozoites (Fig. 3A), meanwhile in treated parasites, the presence of actin was observed in patches and it was also detected on the surface of cysts (Fig. 3B, C). It is worth mentioning that actin co-localised with CWP-1 in some ESVs of treated parasites (Fig. 3H) and on the surface of cysts (Fig. 3I). Control parasites did not stain for CWP-1 (Fig. 3D, G).

**Fig. 4:**
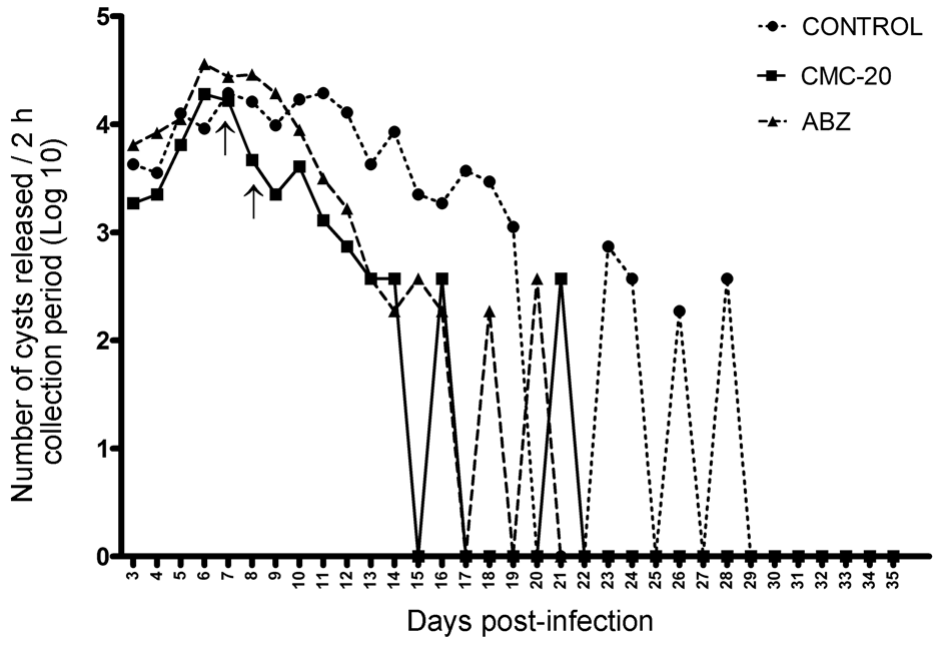
number of cysts released in the feces of non-treated *Giardia muris* infected mice or infected mice treated with **CMC-20** or albendazole (ABZ). BALB/c mice were orally infected with 1 x 10^3^ *G. muris* cysts and treated with **CMC-20** or ABZ at 50 mg/kg/day at days 7 and 8 after infection (arrows). Each point represents the number of cysts (Log_10_) released per 2 h collection period of six animals from days 3 to 35 pi.

*Evaluation of the in vivo giardicidal activity of CMC-20* - The giardicidal activity of CMC-20 was evaluated in BALB/c mice experimentally infected with *G. muris*. CMC-20 at 50 mg/kg/day was administered intragastrically at days seven and eight pi. Non-treated infected mice and infected mice treated with ABZ (50 mg/kg/day) were included as controls (Fig. 4). The release of cysts in feces of non-treated infected mice (Fig. 4 -●-) was observed till day 29 pi, however, intermittent release was detected from day 22 till day 28 pi. No cysts release was observed from day 29 to 35 pi. On the other hand, in infected animals treated with CMC-20 (Fig. 4 -■-), the number of cysts released in feces was drastically reduced as well as the infection time in comparison to the infection control. Cyst load was reduced immediately after the first administration of CMC-20, decreasing from 16473 cysts at day seven pi to 4627 cysts at day eight pi. At day 15 pi no cysts were detected, however, at day 16 and 21 pi an intermittent release of cysts was observed, reaching a maximum release of 370 cysts. From day 22 pi until day 35 pi no cyst release was detected. In infected mice treated with ABZ (Fig. 4 -▲-), the release of cysts started to decrease after day 8 pi (28877 cysts) and no cysts in feces were detected at day 17 pi, however, at day 18 and 20 pi an intermittent release of cysts was observed (185 and 370 cysts, respectively). Cyst release was not detected from day 21 pi up to day 35 pi.

## DISCUSSION

There are few different classes of drugs available for treatment of human giardiasis, and therapeutic failure occurs frequently, being drug resistance the most important cause. Metronidazole, a nitroimidazole derivative, is commonly used for first-line treatment of human giardiasis; however, cases of drug-resistance have been frequently reported.^7^ A prevalence of metronidazole-resistant clinical isolates was up to 20% in people attended in a travel clinic in Spain, especially those with infections acquired in Asia.^19^ Besides, metronidazole treatment failure increased from 15.1% in 2008 to 40.2% in 2013 in London, England.^7^ ABZ, a benzimidazole derivative, and NTZ, a nitrothiazole derivative, are alternatively used in the treatment of giardiasis. Regarding ABZ, treatment failure has also been reported^20^ and ABZ resistance has been described in clinical isolates.^14^ In relation to NTZ, clinical cure was demonstrated in only 50% of nitroimidazole refractory cases treated with NTZ^7^ and more recently, a case of treatment failure with NTZ was reported; and NTZ resistance was confirmed in the clinical isolate (N1-INP).^13^ Therefore, development of new giardicidal drugs remains urgent, in particular given the disadvantages of the available drugs mentioned above.

Then, in this study it was of interest to investigate the activity of **CMC-20,** a hybrid molecule of NTZ and *N*-methyl-1*H-*benzimidazole, against *G. intestinalis* strains with different susceptibility or resistance to the reference drugs, ABZ and NTZ. Data obtained showed that NTZ was more active than **CMC-20** and showed the highest SI against the chronic and acute clinical isolates as well as the ABZ resistant strain. Nevertheless, **CMC-20** showed IC_50_ values in the nanomolar order against all tested strains; and importantly, it showed 3 and 28 times higher activity against ABZ and NTZ resistant strains than ABZ and NTZ, respectively. Besides, **CMC-20** showed two and 23 times higher selectivity against the ABZ and NTZ resistant strains than ABZ and NTZ, respectively. It is important to point out that, although NTZ was highly active and selective against parasite strains susceptible to this drug, resistance to NTZ as well as ABZ has been reported not only on experimentally induced resistant strains,^11^ ^,^ ^12^ but also in clinical isolates^13^ ^,^ ^14^ and treatment failure has been documented.^7^ ^,^ ^20^ In this regard, **CMC-20** could have a potential use in the treatment of giardiasis.

Previous proteomic analysis of *G. intestinalis* WB strain treated with **CMC-20** revealed changes on the expression of alpha and beta tubulin as well as some proteins involved in the encystment process,^16^ but it was not further characterised. Herein, IFI studies, demonstrated that **CMC-20** induced changes not only on the distribution of the cytoskeletal proteins, alpha and beta tubulin, but also of the post-translationally modified acetylated and tyrosinated-alpha tubulin. These cytoskeletal proteins were localised, in agreement with other studies,^21^ ^,^ ^22^ in structures such as the ventral disc, flagella, median body and basal bodies of the control parasites. Significantly, these proteins were not detected in the median body of around 80% of the **CMC-20** treated trophozoites. The median body is considered to be a microtubule reservoir that participates in the ventral disc progenesis.^21^ Interestingly, previous FESEM studies performed after membrane detergent extraction of **CMC-20** treated parasites showed the ventral disc spiral disorganised and the bare area expanded. Besides, proteomic analysis demonstrated that **CMC-20** down-regulated the expression of alpha and beta tubulin.^16^ These observations together with the absence of the median body in treated parasites, observed by IFI, indicate that **CMC-20** affects the parasite cytoskeletal proteins. The absence of the median body has also been shown in *Giardia* trophozoites treated with drugs that affect the parasite microtubules, such as ABZ and nocodazole.^8^ ^,^ ^23^

On the other hand, the presence of tyrosinated-alpha tubulin but not detyrosinated- alpha tubulin, can be explained based on the examination of the *Giardia* Genome Project.^24^ It revealed the presence of a gene (Contig 725, ORF 14498) with a high degree of similarity to other tubulin tyrosine ligase family genes, and the predicted protein sequence contains a canonical tubulin tyrosine ligase, However, the examination of C terminal fragments of alpha tubulin showed complete retention of the terminal tyrosine indicative of the absence of a tubulin carboxypeptidase.^24^ In addition, it is known that acetylated-alpha tubulin forms more stable microtubules, meanwhile tyrosinated-alpha tubulin is present in more dynamic microtubules, then, *G. intestinalis* cytoskeleton seems to be constituted by both types of microtubules.

The presence of CWP-1 in ESVs and on the surface of **CMC-20** treated parasites was shown by IFI assays, confirming that **CMC-20** triggers the parasite encystation. It is worth mentioning that although **CMC-20** induces some cells to encyst (around 10%), previous FESEM studies of parasites treated with **CMC-20** at 0.010 μM and 7.8 μM showed the cysts surface severely damaged,^16^ suggesting that they may be unviable. It may be that the stress conditions induced by **CMC-20**, severely affect the trophozoite survival, but only some of them can undergo the encystment process, although the cysts surface is damaged. ABZ and some benzimidazole derivatives also induce the encystment process in *Giardia*.^25^

The localisation of alpha-7.2 giardin associated to the plasma membrane of trophozoites was also shown by Weiland et al.^26^ In this regard, the association of several alpha-giardins (alpha-1, alpha -2, alpha-7.3, alpha -8 and alpha-11) to the plasma membrane has been demonstrated.^26^ ^,^ ^27^ It was proposed that alpha giardins may stabilise the cytoskeleton by crosslinking the plasma membrane with the parasite microtubules.^26^ For the first time, the co-localisation of CWP-1 with alpha-7.2 giardin in ESVs and on the surface of parasites during the encystment process was demonstrated. In previous studies of low-density ESVs fractions, the presence of alpha-7 giardin was detected by two-dimensional electrophoresis and mass spectrometry analysis, however, it was considered as a contamination during ESVs purification.^28^ The association of alpha-7.2 giardin with CWP-1, during parasite encystation, requires further studies. Moreover, the co-localisation of actin with CWP-1 in some ESVs was also observed. In this regard, Paredez et al.^29^ described the recruitment of actin to mature ESVs during the encystment process and Castillo-Romero et al.^30^ demonstrated that Rab11 and actin are involved in *Giardia* encystation.

The *in vivo* giardicidal activity of **CMC-20** was shown in an experimental model of giardiasis. **CMC-20** induced an important reduction in the cyst load as well as in the infection time in comparison to non-treated infected mice. It is worth to mention that the giardicidal activity of **CMC-20** was similar to that shown by ABZ.

Herein, the broad activity and high selectivity of **CMC-20** against isolates from patients with chronic or acute giardiasis as well as ABZ and NTZ resistant strains was demonstrated. Indeed, the effect of **CMC-20** on the parasite microtubule reservoir, demonstrated by IFI assays, contributes to its anti-*Giardia* activity. The co-localisation of alpha-7.2 giardin and actin with CWP-1 during the encystment process induced by **CMC-20** needs further studies. **CMC-20** reduced the infection time and cyst load in feces of *Giardia* infected mice similar to ABZ. Further pharmacokinetic and toxicological studies, among others are required in order to confirm the potential use of **CMC-20** in the treatment of giardiasis.
