# Supplementary figures and images for: Characterisation of the in vitro activity of a Nitazoxanide-N-methyl-1H-benzimidazole hybrid molecule against albendazole and nitazoxanide susceptible and resistant strains of Giardia intestinalis and its in vivo giardicidal activity
Source: Mem Inst Oswaldo Cruz. 2020 Feb 7;115:e190348. doi: 10.1590/0074-02760190348 (PMC7012584; doi:10.1590/0074-02760190348)

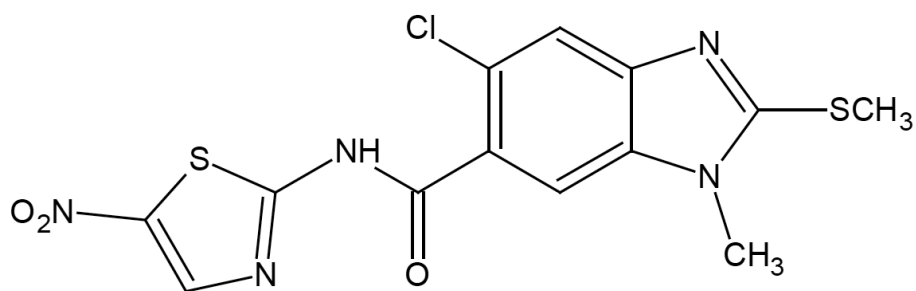

Compound **CMC-20**: 5-chloro-1-methyl-2-(methylsulfanyl)-*N*-(5-nitro-1,3-thiazol-2-yl)-1*H*-benzimidazole-6-carboxamide.

Supplement: Supplementary file 1 [file 1678-8060-mioc-115-e190348-s.pdf]
